# CaMKII/proteasome/cytosolic calcium/cathepsin B axis was present in tryspin activation induced by nicardipine

**DOI:** 10.1042/BSR20190516

**Published:** 2019-07-02

**Authors:** Juan Xiao, Houmin Lin, Binggang Liu, Junfei Jin

**Affiliations:** 1 Laboratory of Hepatobiliary and Pancreatic Surgery, the Affiliated Hospital of Guilin Medical University, Guilin 541001, Guangxi, People’s Republic of China; 2 Department of Surgery, Xiangya Hospital, Central South University, Changsha 410008, Hunan, People’s Republic of China; 3 China-U.S.A. Lipids in Health and Disease Research Center, Guilin Medical University, Guilin 541001, Guangxi, People’s Republic of China; 4 Guangxi Key Laboratory of Molecular Medicine in Liver Injury and Repair, Guilin Medical University, Guilin 541001, Guangxi, People’s Republic of China

**Keywords:** CAMKII, cathepsin B, cytosolic calcium, nicardipine, proteasome inhibition, Trypsinogen activation

## Abstract

Premature trypsinogen activation is the early event of acute pancreatitis. Therefore, the studies on the processes of trypsinogen activation induced by compounds are important to understand mechanism underly acute pancreatitis under various conditions. Calcium overload in the early stage of acute pancreatitis was previously found to cause intracellular trypsinogen activation; however, treatment of acute pancreatitis using calcium channel blockers did not produced consistent results. Proteasome activity that could be inhibited by some calcium channel blocker has recently been reported to affect the development of acute pancreatitis; however, the associated mechanism were not fully understood. Here, the roles of nicardipine were investigated in trypsinogen activation in pancreatic acinar cells. The results showed that nicardipine could increase cathepsin B activity that caused trypsinogen activation, but higher concentration of nicardipine or prolonged treatment had an opposite effect. The effects of short time treatment of nicardipine at low concentration were studied here. Proteasome inhibition was observed under nicardipine treatment that contributed to the up-regulation in cytosolic calcium. Increased cytosolic calcium from ER induced by nicardipine resulted in the release and activation of cathepsin B. Meanwhile, calcium chelator inhibited cathepsin B as well as trypsinogen activation. Consistently, proteasome activator protected acinar cells from injury induced by nicardipine. Moreover, proteasome inhibition caused by nicardipine depended on CaMKII. In conclusion, CaMKII down-regulation/proteasome inhibition/cytosolic calcium up-regulation/cathepsin B activation/trypsinogen activation axis was present in pancreatic acinar cells injury under nicardipine treatment.

## Introduction

Premature trypsinogen activation and calcium overload are early events of acute pancreatitis characterized as self-digestion and might cause multiple organ dysfunctions in severe cases [1,2].

Mechanism detection for trypsinogen activation in pancreatic acinar cells is very important for the understanding of acute pancreatitis pathogenesis or the development of the associated therapy [3,4] since intra-trypsinogen activation is the important events in acute pancreatitis [5]. *PRSS1* and *PRSS2* encode the most abundant forms of trypsinogen [6]. Genetic polymorphisms analysis showed that the C allele at the loci of *PRSS1* and *PRSS2* were significantly correlated to acute pancreatitis in male patients with alcohol abuse and smoking [7]. Besides, mutations of *SPINK1* and *CTRC* were directly associated with acute pancreatitis [8,9]. Of note, the former inhibited trypsin, and the latter had dual effect [10–12]. Except the genetic approach trypsin activity can also be influenced by chemical reagents. In the well-known acute pancreatitis animal model induced by cerulein, L-arginine, or sodium taurocholate, all three indicated metabolite derivatives lead to premature trypsinogen activation [13,14]. These chemical molecules were widely investigated for determination of the mechanism underlying trypsinogen activation or drug design for acute pancreatitis. However, cases in which trypsin activity is regulated by various types of compounds have not been fully investigated.

As it is well understood that calcium overload promotes cellular trypsinogen activation [15,16]. Inhibition of cytosolic calcium by the chelator BATAP AM indeed decreased trypsin activity [14]. Besides, in previous studies, the effects of some types of voltage-gated calcium channel blockers on acute pancreatitis were evaluated, and a few of them showed protective roles [17,18]. However, a previous study also found that continuous exposure to voltage-dependent calcium channel blockers induced acute pancreatitis in mice [19]. In all, the actual roles of the voltage-gated channels blockers and the associated mechanism have not been clarified in acute pancreatitis.

Previous reports found that cathepsin B, which is located in the lysosome, could recognize and then activate trypsinogen when the lysosome was fused with zymogen granules [20–22]. Furthermore, cathepsin B inhibition through a genetic approach or by using chemical reagents decreased the activity of trypsin [4,23]. Increased cytosolic calcium could promote cathepsin B activation, which was related to the damage in lysosome integrity in cancer or brain cells [24,25]. However, the details for cytosolic calcium working on cathepsin B have not been fully investigated in pancreatic acinar cells.

Proteasome is well known to degrade unneeded or damaged protein that is beneficial for cell survival [26]. Proteasome inhibitors, bortezomib and ixazomib, approved by FDA in the United States that have been used in clinic for multiple myeloma therapy were recently reported to cause acute pancreatitis [27–29]. Meanwhile, some L-type calcium channel blockers showed the ability to cause proteasome inhibition [30]. Until now, the mechanism underlying the adverse effect of these two drugs has not been understood.

In the present study, we investigated the effect and underlying mechanisms of the voltage-gated channel blocker, nicardipine, on trypsinogen activation in rat pancreatic acinar cell lines AR42J and mouse primary pancreatic acinar cells.

## Materials and methods

### Chemicals and antibodies

The following antibodies were used: anti-cathepsin B (rabbit, CST, 31718S), anti-actin (mouse, PTG, 60008-1-Ig), anti-Ubiquitination (rabbit, PTG, 10201-2-AP), anti-Flag (mouse, Sigma, F1804), and anti-tubulin (rabbit, PTG, 10094-1-AP). Cerulein, nicardipine, CA074me, and BAPTA AM were obtained from MCE. Fluo-4 AM was obtained from Thermo Fisher. Sulforaphane was obtained from Sigma-Aldrich. GSK-7975A was obtained from MCE.

### Cell culture

AR42J (exocrine pancreatic tumor cells, ATCC) was cultured in Dulbecco’s modified Eagle’s medium (DMEM, Gibco) containing 10% fetal bovine serum (FBS, Gibco) and 1% penicillin–streptomycin (Solabal). All cultures were maintained in a 37°C incubator with 5% CO_2_.

### Primary pancreatic acinar cells isolation

Pancreas tissue were separated from C57BL/6 mice, and collagenase was used to digest pancreatic acinar cells. After filtration, primary pancreatic acinar cells were cultured in DMEM (Gibco) supplemented with 10% FBS and used for subsequent experiments. All animal care and experimental procedures were approved by the Ethical Committee on Animal Experiments at Guilin Medical University and the National Institutes of Health guide for the care and use of Laboratory animals (NIH Publications No. 8023, revised 1978)

### Compounds treatment and protein overexpression

Time course effect experiments: 2.5 μM nicardipine were added in AR42J cells or isolated pancreatic acinar cells for indicated durations. The dose course experiments: Nicardipine, at indicated concentrations, was added in AR42J cells for 6 h or in isolated pancreatic acinar cells for 60 min. Nicardipine plus compound (2 μM CA074me, 10 μM BAPTA AM, 10 μM Sulforaphane or 10 μM GSK-7975A) treatment in AR42J cells: 2.5 μM nicardipine was added in AR42J cells for 6 h during which indicated compound was added (CA074me for 3 h, BAPTA AM for 6 h, Sulforaphane for 6 h or GSK-7975A for 3 h). Nicardipine plus compound (2 μM CA074me, 10 μM BAPTA AM, 10 μM Sulforaphane or 10 μM GSK-7975A) treatment in isolated pancreatic acinar cells: 2.5 μM nicardipine was added in cells for 1 h during which indicated compound was added (CA074me for 0.5 h, BAPTA AM for 1 h, Sulforaphane for 1 h or GSK-7975A for 0.5 h).

Overexpression: AR42J cells (isolated pancreatic acinar cells) were infected with control vector or CaMKII expression plasmids containing lentivirus for 48 h, and then 2.5 μM nicardipine was added for additional 6 h (1 h in isolated pancreatic acinar cells).

### Cell survival, apoptosis, and necrosis assay

Cells were seeded in the 96-well culture plate and nicardipine at indicated concentration were added into cells for indicated durations. Then, cells were incubated with CCK-8 (Dojindo) reagent for 1 h, and the cell viability was measured by microplate reader. For apoptosis and necrosis assay, nicardipine-treated cells were subjected to apoptosis assay by using caspase 3/7 Glo kits (promega) and necrosis (LDH release) by using Cytotox-96 cell cytotoxicity kit (Pierce).

### Trypsin activity assay

AR42J cells were lysed, each sample was incubated with butoxycarbonyl-Gln-Ala- Arg-7-amido-4-methylcou-marin hydrochloride (Sigma), in trypsin reaction buffer (10 mM Tris, 20 mM CaCl_2_, pH 7.4) at 37°C for 30 min. The trypsin activity characterized as fluorescence intensity of trypsin substrate was measured (excitation: 380 nm, emission: 450 nm) in a fluorescence microplate reader [31].

### Cathepsin B activity assay

Cathepsin B activity was measured according to the Enzymatic Assay of Cathepsin B protocol provided by sigma Inc. Briefly, cell lysates were incubated with cathepsin B substrate Nα–CBZ–Arg–Arg–7–amido–4–methylcoumarin at 37°C, and the fluorescence intensity was determined at 440 nm in a fluorescence microplate reader under excitation at 348 nm.

### Cytosolic calcium content measurement

After indicated treatments, cells were incubated with 1 μM Furo-4 AM/HBSS at 37°C for 10 min. Cells were washed with HEPES buffer and detached. Fluorescence intensity of cells in HEPES buffer was detected by FACS (Attune® NxT, life technologies) under excitation at 488 nm or cells were imaged by fluorescence microscopy.

### Western blotting

The protein mixture in lysates from AR42J cells or animal pancreas were separated by SDS-polyacrylamide gel electrophoresis (PAGE) and transferred to PVDF membranes (Millipore). The membranes were incubated with the indicated primary antibodies, then with anti-rabbit or anti-mouse IgG secondary antibodies (PTG), the reactions were visualized by chemiluminescence reagents.

### Immunofluorescence staining

Cells were fixed with 4% paraformaldehyde, permeated with 0.3% Triton x-100, and blocked with 5% goat serum at room temperature for 30 min, followed by incubation with anti-cathepsin B antibody at 4°C overnight. Finally, cells were incubated with FITC-conjugated rabbit secondary antibody (Abcam) at room temperature for 1 h. The distribution of cellular cathepsin B was visualized by fluorescence microcopy (400×).

### Statistical analysis

Error bars for enzyme activity or intensity of calcium specific staining were presented as the standard deviation of triplicate samples. Error bars for Western blot analysis represent the standard deviation among densitometry data from three unique experiments. One-way ANOVA was used as statistical analysis by using SPSS. * or # *P* < 0.05, ** or ## *P* < 0.01, *** or ### *P* < 0.001.

## Results

### Nicardipine increases trypsin activity in pancreatic acinar cells

**Figure 1:**
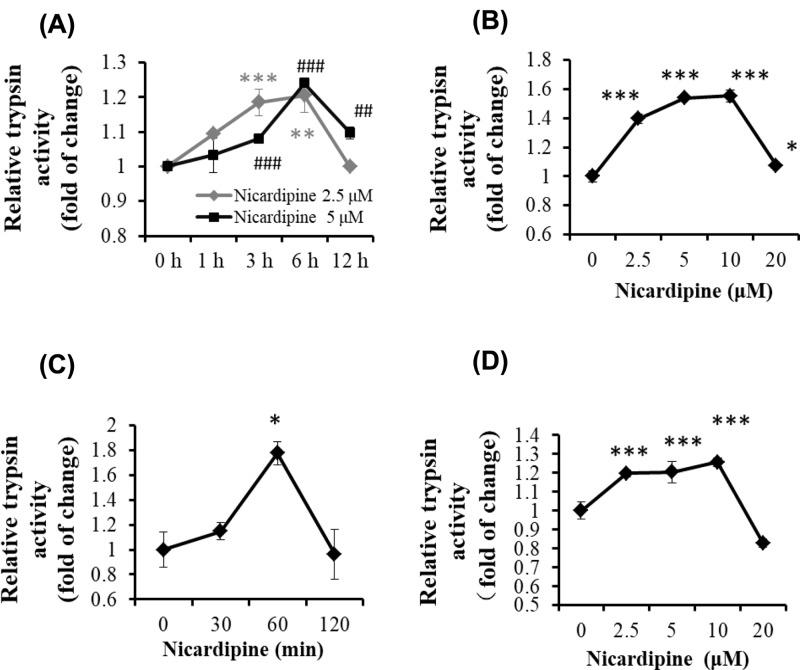
Nicardipine induces trypsinogen activation in pancreatic acinar cells AR42J cells were treated with 2.5 or 5 μM nicardipine for 0, 1, 3, 6, and 12 h (**A**) or with nicardipine at indicated concentrations for 6 h (**B**), and then cells were lysed, and cell lysates were subjected to trypsin activity assays. Pancreatic cells were isolated from C57BL/6 mice and cultured in DMEM media. Primary cells were treated with 2.5 μM nicardipine for indicated durations (**C**) or with nicardipine at indicated concentrations for 1 h (**D**), and then cells were lysed and subjected to trypsin activity assay. Error bars for enzyme activity were presented as the standard deviation of triplicate samples.VS 0h, * *P* < 0.05, ** or ## *P* < 0.01, *** or ### *P* < 0.001.

Calcium overload occurs in the initiation of acute pancreatitis, and previous studies have demonstrated that up-regulation of cytosolic calcium promoted trypsinogen activation in pancreatic acinar cells. As a type of L-type channel blocker, nicardipine has the potential to regulate the activity of trypsin in pancreatic acinar cells. Therefore, we chose the rat exocrine pancreatic tumor cell line AR42J that possesses L-type calcium channel to evaluate the relationship between nicardipine and trypsinogen activation. Surprisingly, when AR42J cells were treated with nicardipine, cellular trypsinogen activation was observed. Specifically, nicardipine increased the activity of trypsinogen in a time- and a dose-dependent manner (Figure 1A,B). Moreover, we found that trypsin activity was on a downward trend in AR42J cells with prolonged treatment (12 h) or higher concentration (20 μM) of nicardipine. We therefore focused on changes in trypsin activity induced by the low concentration of nicardipine for short time periods in the next investigation.

Next, we tested the effect of nicardipine in primary pancreatic acinar cells that were isolated from C57BL/6 mice and did not present L-type calcium channel. Similarly, nicardipine caused a temporary elevation of trypsin activity at 1 h in primary acinar cells (Figure 1C). Compared with the results in AR42J cells, dose-dependent effect of nicardipine on trypsin activity in primary cells was not very obvious. As shown in Figure 1D, the fluctuation of the nicardipine concentration (2.5, 5, and 10 μM) did not lead to the significant changes in trypsin activity. When higher concentration (20 μM) of nicardipine was used, trypsin activity decreased, which was consistent with the phenomenon in AR42J cells (Figure 1B). It is needed to be mentioned here that nicardipine did not showed significant cytotoxicity in our study (Supplementary Figures S1–S3).

Taken together, these results suggested that nicardipine caused transient stimulation in trypsin activity in pancreatic acinar cells in an L-type calcium channel independent manner. The associated mechanisms were investigated next.

### Nicardipine activates trypsinogen through cathepsin B in AR42J cells

**Figure 2:**
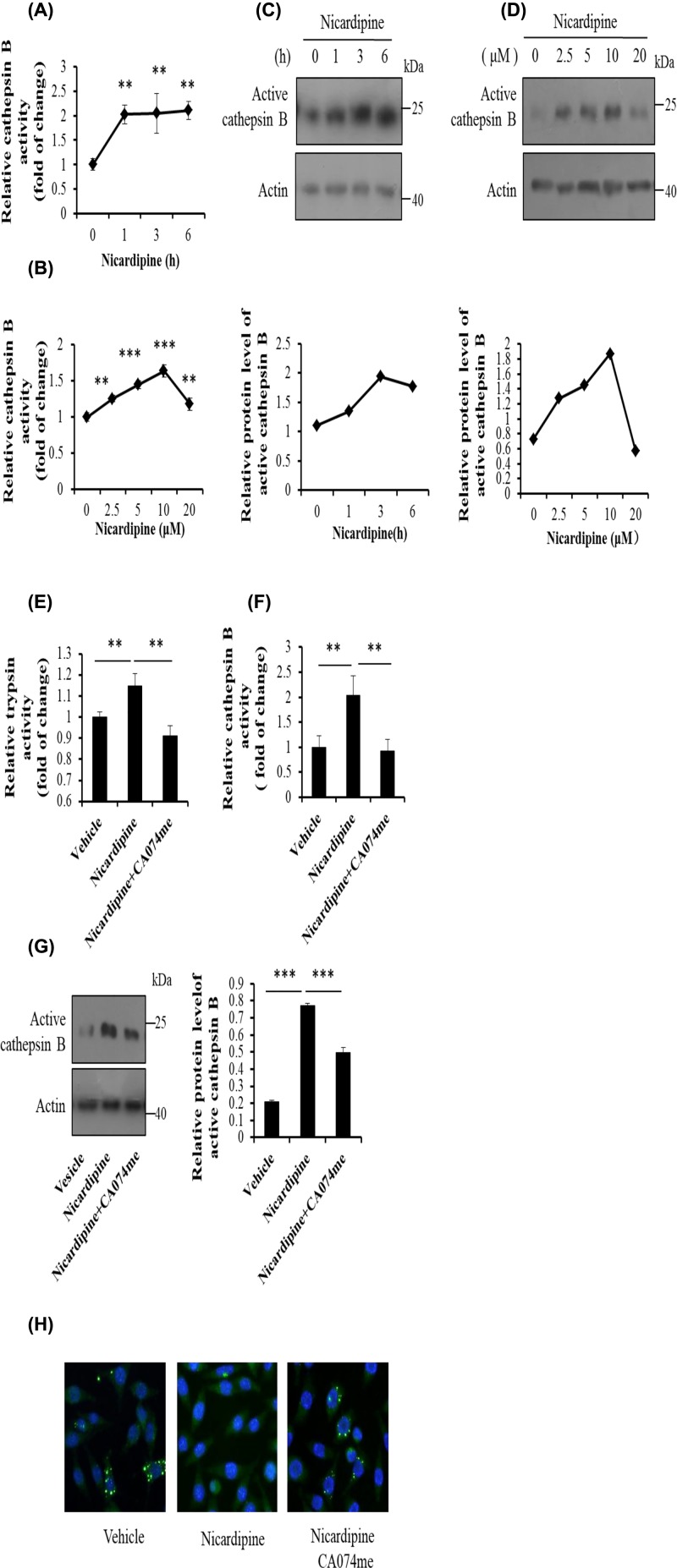
Trypsinogen activation is mediated by cathepsin B under nicardipine treatment in AR42J cells AR42J cells were incubated with 2.5 μM nicardipine for indicated durations (**A** and** C**) or with nicardipine at indicated concentrations for 6 h (**B** and **D**). The cells were then lysed, and cell lysates were subjected to cathepsin B activity assay (A and B) or Western blotting using anti-cathepsin B antibody, with actin as a loading control (C and D). Error bars for enzyme activity were presented as the standard deviation of triplicate samples; **P < 0.01 and ***P < 0.001. The graphs below the blots were quantified using ImageJ as means of two independent sets of experiments. AR42J cells were treated with 2.5 μM nicardipine for 6 h during which cathepsin B inhibitor CA074me (2 μM) were absent or present for 3 h. Cells were lysed, cell lysates were subjected to trypsin (**E**) or cathepsin B (**F**) activity assay or Western blotting using anti-cathepsin B antibody, with actin as a loading control (**G**). Error bars for enzyme activity were presented as the standard deviation of triplicate samples; **P < 0.01. The graphs on the right of the blotting provided are quantified using ImageJ as means ± SD of three independent sets of experiments; ***P < 0.001. After indicated treatments, AR42J cells were fixed and then were subjected to the immunofluorescence assay using anti-cathepsin B antibody. Then, cells were imaged with fluorescence microscopy (**H**).

Cathepsin B is a well-known promoter for trypsinogen activation. Since nicardipine could induce intracellular trypsin, we sought to determine whether this compound could increase the activity of trypsin through cathepsin B. In order to figure out the role of cathepsin B in nicardipine stimulated trypsinogen activation, first we treated AR42J cells with nicardipine at different concentrations or for different durations. As we expected, nicardipine increased the activity of cathepsin B (Figure 2A,B). Besides, both the time and dose response curve for cathepsin B activity were matched with that for trypsin activity (Figure 1A,B). Meanwhile, cleaved cathepsin B which reflects the activity of itself was measured by Western blotting. The protein levels of active cathepsin B were significantly increased under nicardipine (low concentration) treatment for indicated durations (Figure 2C,D). These results suggested that cathepsin B was probably related to the up-regulation of cellular trypsin under nicardipine treatment.

AR42J cells were then treated with cathepsin B inhibitor CA074me in the presence of nicardipine. We found that CA074me inhibited the elevation in trypsin activity induced by nicardipine (Figure 2E). The inhibition effect of CA074me on cathepsin B was confirmed by cathepsin B activity assay and Western blotting analysis for the protein levels of its active form (Figure 2F,G).

A previous study reported that cathepsin B activated trypsinogen in zymogen and then leaked into cytosol triggered by trypsinogen [32]. To investigate how the nicardipine regulated cathepsin B in detail, immunofluorescence staining was performed. As shown here, compared with the control, nicardipine caused the diffuse distribution of cathepsin B while CA074me reversed it (Figure 2H). The results indicated that nicardipine might influence the location of cathepsin B.

Taken together, these data suggested that nicardipine induced trypsin in AR42J cells via cathepsin B.

### Nicardipine activates trypsinogen through cathepsin B in primary pancreatic acinar cells

**Figure 3:**
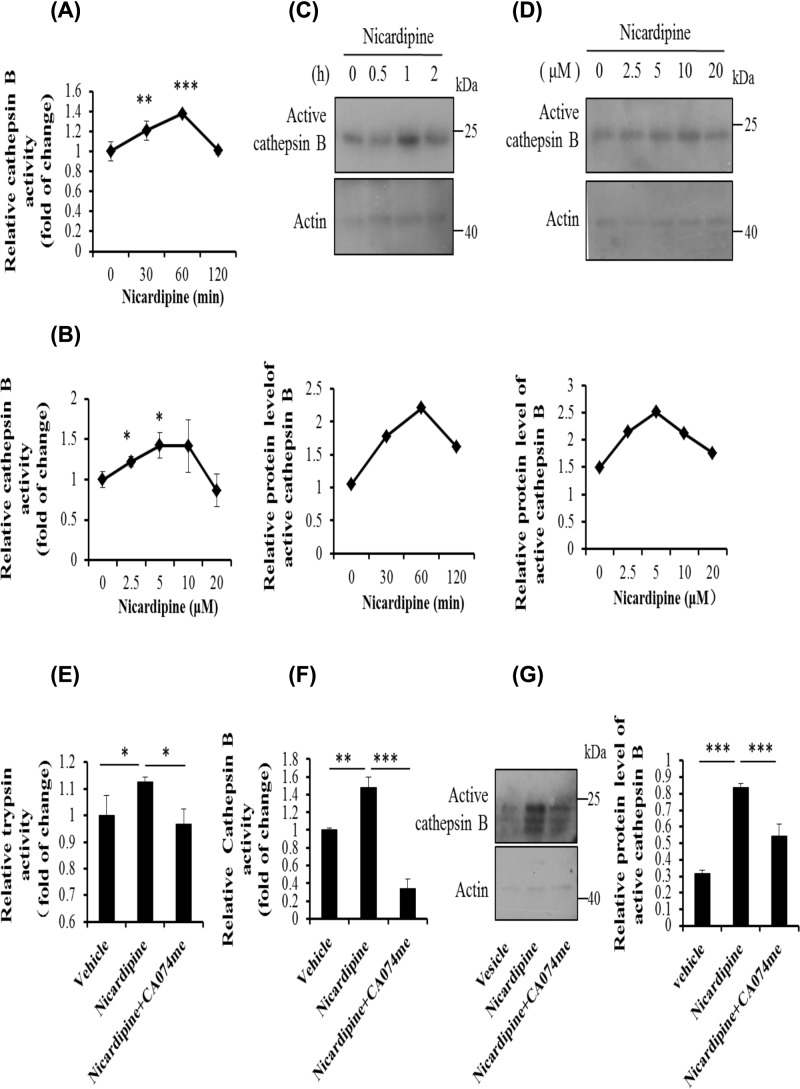
Trypsinogen activation is mediated by cathepsin B under nicardipine treatment in primary pancreatic acinar cells Primary pancreatic acinar cells were incubated with 2.5 μM nicardipine for indicated durations (**A** and **C**) or with nicardipine at indicated concentrations for 1 h (**B** and **D**). The cells were lysed, cell lysates were subjected to cathepsin B activity assay (A and B) or Western blotting using anti-cathepsin B activity, with actin as a loading control (C and D). Error bars for enzyme activity were presented as the standard deviation of triplicate samples. *P < 0.05, **P < 0.01, and ***P < 0.001. The graphs below the blots were quantified using ImageJ as means of two independent sets of experiments. Primary cells were treated with 2.5 μM nicardipine for 1 h with or without cathepsin B inhibitor CA074me (2 μM). Cells were lysed, cell lysates were subjected to trypsin (**E**) or cathepsin B (**F**) activity assay or Western blotting using anti-cathepsin B antibody, with actin as a loading control (**G**). Error bars for enzyme activity were presented as the standard deviation of triplicate samples. *P < 0.05, **P < 0.01 and ***P < 0.001. The graphs on the right of the blotting provided are quantified using ImageJ as means ± SD of three independent sets of experiments; ***P < 0.001.

Cathepsin B activity was also detected in isolated mouse pancreatic acinar cells. Time response for cathepsin B activity was in accordance with trypsin activity in AR42J cells (Figure 3A). However, slightly different from the effect of nicardipine on trypsin, the fold changes of the amount of nicardipine did not result in the significant changes in cathepsin B activity (Figure 3B). Similar results were obtained from the Western blotting analysis (Figure 3C,D). As expected, CA074me blocked the effect of nicardipine on trypsin, and cathepsin B activity which were confirmed by enzyme activity assay and Western blotting analysis (Figure 3E–G).

### Nicardipine-mediated up-regulation in cathepsin B activity depends on cytosolic calcium in AR42J cells

**Figure 4:**
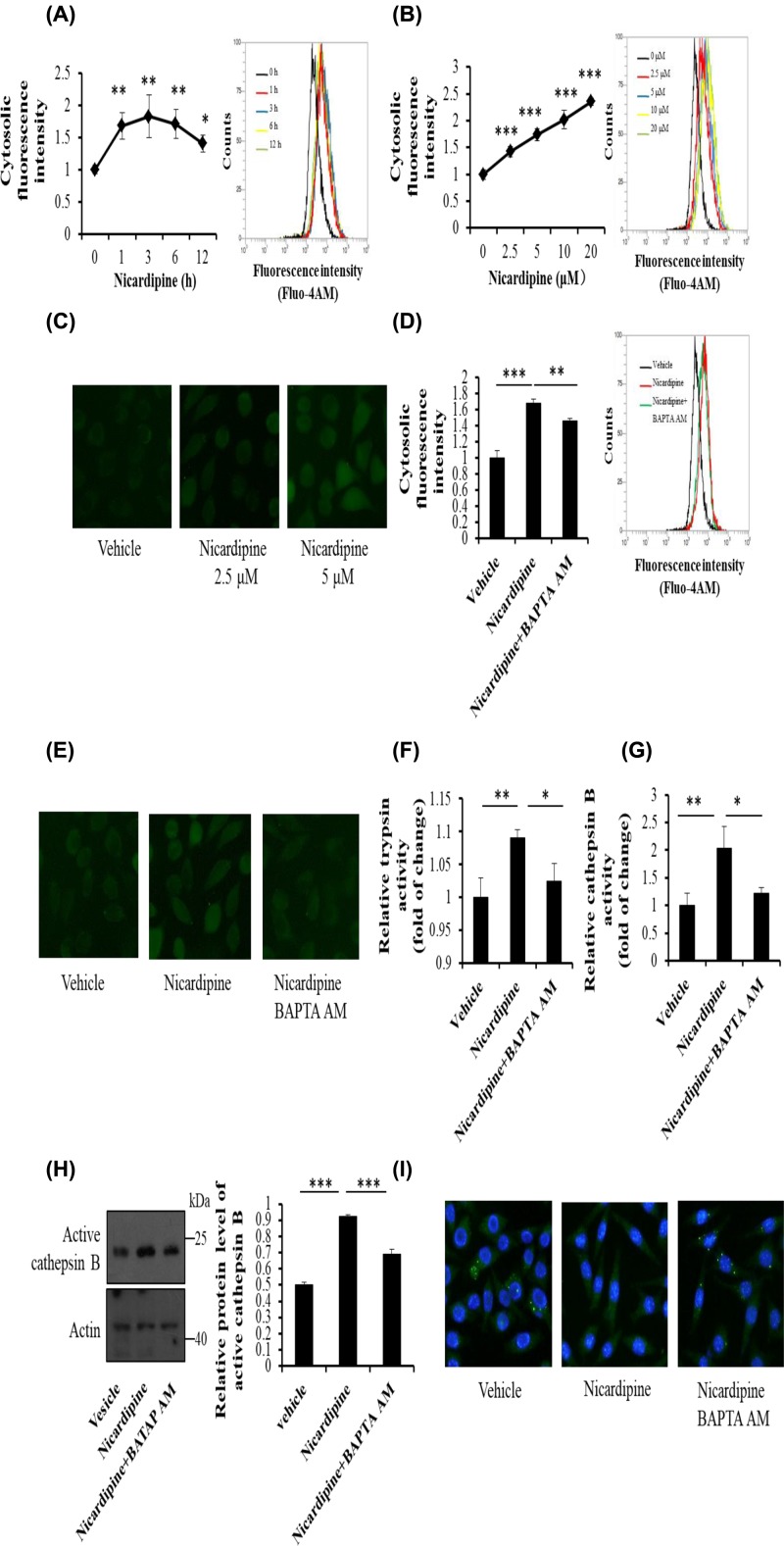
Nicardipine-induced trypsinogen and cathepsin B activation depends on cytosolic calcium in AR42J cells AR42J cells were incubated with 2.5 μM nicardipine for indicated durations (**A**) or with nicardipine at indicated concentrations for 6 h (**B** and** C**). Cells were stained with calcium specific dye fluo-4AM (1 μM) at 37°C for 10 min, and then were subjected to calcium intensity assay by FACS after digestion (A and B) or were directly imaged with fluorescence microscopy (C). AR42J cells were incubated with 2.5 μM nicardipine for 6 h with or without calcium chelator BAPTA AM (10 μM). Cells were stained with calcium-specific dye fluo-4AM (1 μM) at 37°C for 10 min, and then were subjected to calcium intensity assay by FACS (**D**) or were directly imaged with fluorescence microscopy (**E**). After the indicated treatment, cells were lysed and the cell lysates were subjected to trypsin (**F**) and cathepsin B (**G**) activity assay or were subjected to Western blotting analysis using anti-cathepsin B antibody, with actin as the loading control (**H**). Error bars for enzyme activity or intensity of calcium specific staining were presented as the standard deviation of triplicate samples. Error bars for graph of Western blot analysis represent the standard deviation between densitometry data from three unique experiments; *P < 0.05, ** *P* < 0.01 and ***P < 0.001. AR42J cells under indicated treatment were fixed and then were subjected to the immunofluorescence assay using anti-cathepsin B antibody, and the cells were imaged with fluorescence microscope (**I**).

It has been reported that cytosolic calcium overload caused co-localization between zymogen and lysosome when trypsinogen was activated by cathepsin B [20–22]. Since we demonstrated that nicardipine induced trypsin through cathepsin B, together with the fact that calcium up-regulation induced cathepsin B in some other types of cell [24,25], we supposed the mechanism underlying the indicated enzyme activation was related to calcium overload. To validate this hypothesis, nicardipine was added to AR42J cells and then cytosolic calcium was detected with a calcium-specific dye. After staining and qualified by FACS, AR42J cells treated with nicardipine showed much higher fluorescence intensity than control (Figure 4A,B). Meanwhile cells stained with the calcium-specific dye were imaged with fluorescence microscope, and consistent results were observed (Figure 4C). These data suggested that nicardipine increased the total level of cytosolic calcium. Besides, calcium chelator BAPTA AM diminished the elevation in the amount of cytosolic calcium induced by nicardipine (Figure 4D,E).

To determine the correlation between nicardipine-induced upregulation of cytosolic calcium and cathepsin B or trypsin activation, relative enzyme activity was measured. Using spectrophotometry, we found that BAPTA AM reduced the extent of the elevation in the activity of trypsinogen and cathepsin B (Figure 4F,G). Meanwhile the protein level of active cathepsin B was decreased when AR42J was treated with BAPTA AM in the presence of nicardipine (Figure 4H). Based on the immunofluorescence images, cellular distribution changes of cathepsin B under nicardipine treatment was reversed by BAPTA AM (Figure 4I).

Taken together, our findings indicated that nicardipine increased the level of cytosolic calcium and subsequently stimulated cathepsin B, leading to the production of active trypsin.

### Nicardipine-mediated up-regulation in cathepsin B activity depends on cytosolic calcium in primary pancreatic acinar cells

**Figure 5:**
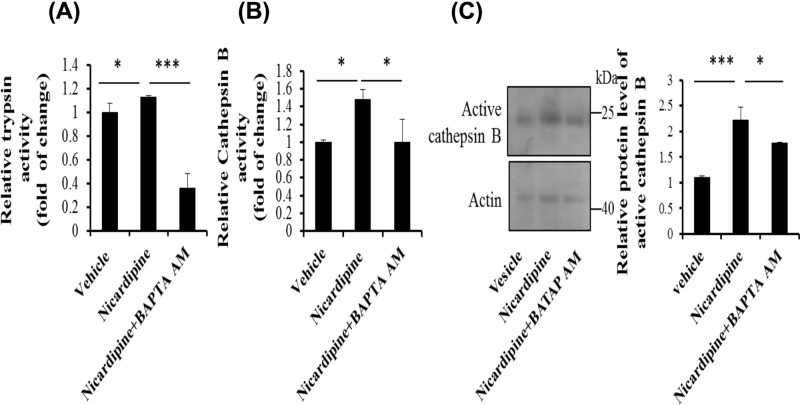
Nicardipine-mediated increase in trypsinogen and cathepsin B activation depends on cytosolic calcium in primary pancreatic acinar cells Primary pancreatic acinar cells were incubated with 2.5 μM nicardipine for 1 h with or without calcium chelator BAPTA AM (10 μM). Cells were lysed and the cell lysates were subjected to trypsin (**A**) and cathepsin B (**B**) activity assay or were subjected to Western blotting analysis using anti-cathepsin B antibody, with actin as a loading control (**C**). Error bars for enzyme activity were presented as the standard deviation of triplicate samples. Error bars for graph of Western blot analysis represented the standard deviation between densitometry data from three unique experiments; *P < 0.05 and ***P < 0.001.

Consistently, when isolated pancreatic acinar cells were incubated with BAPTA AM together with nicardipine, trypsin activity was largely decreased, and cathepsin B activation was significantly inhibited at the same time compared with cells that were only treated with nicardipine (Figure 5A,B). Meanwhile, protein expression of active cathepsin B detected by Western blotting analysis showed that cathepsin B induced by nicardipine was indeed decreased in the presence of BAPTA AM (Figure 5C).

### Nicardipine induced cytosolic calcium overload and trypsinogen activation depended on ER calcium channel store-operated calcium channels

**Figure 6:**
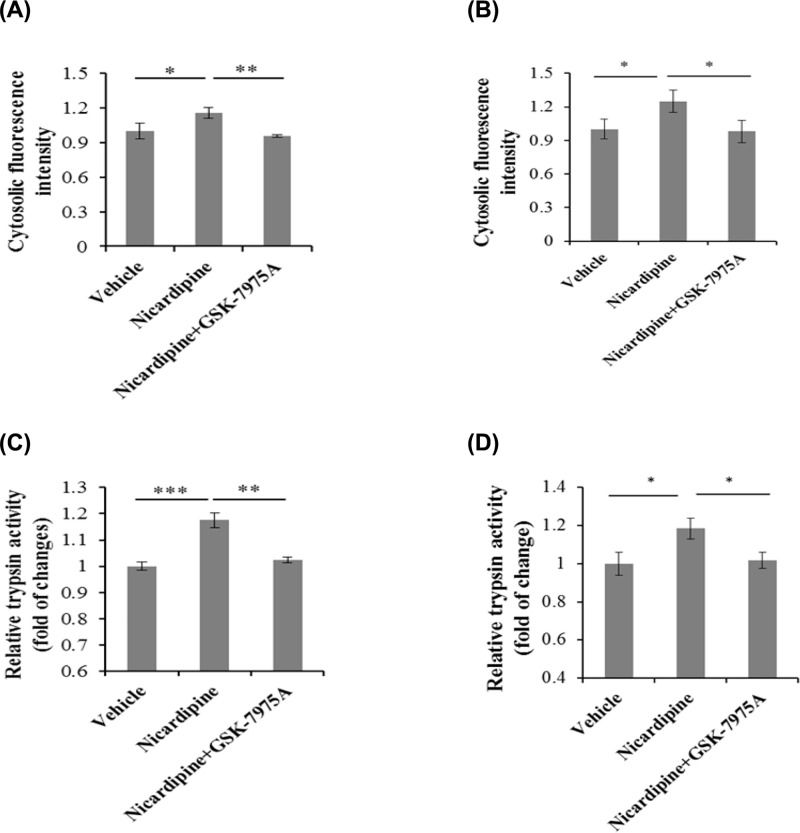
Nicardipine induced cytosolic calcium overload and trypsinogen activation depended on ER calcium channel CRAC (**A** and **C**) 2.5 μM nicardipine was added to AR42J cells for 3 h with or without 10 μM GSK-7975A (CRAC blocker) for additional 3 h. (**B** and **D**) 2.5 μM nicardipine was added to primary acinar cells for 0.5 h with or without 10 μM GSK-7975A (CRAC blocker) for additional 0.5 h. One part of the cells was stained with calcium specific dye Furo-4 AM and then relative calcium content was measured by FACS (A and B); the other part of the cells was lysed and subjected to trypsin activity assay (C and D). The graph shown represented as mean ± SD from three independent experiments. The statistical analysis was performed using one-way ANOVA by SPSS; * *P* < 0.05, ** *P* < 0.01, *** *P* < 0.001.

It was found that nicardipine induced ER stress in our previous research. Therefore, nicardipine-induced cytosolic calcium overload might be from ER calcium channel. GSK-7975A that is the blocker for the well-known calcium channel on ER, store-operated calcium channels (CRAC), was used. The results showed that GSK-7975A decreased cytosolic calcium (Figure 6A,B) as well as trypsinogen activation (Figure 6C,D) induced by nicardipine. Taken together, nicardipine induced calcium overload and trypsinogen premature was mediated by ER calcium channel CRAC.

### Nicardipine induced cytosolic calcium activating trypsinogen through proteasome inhibition

**Figure 7:**
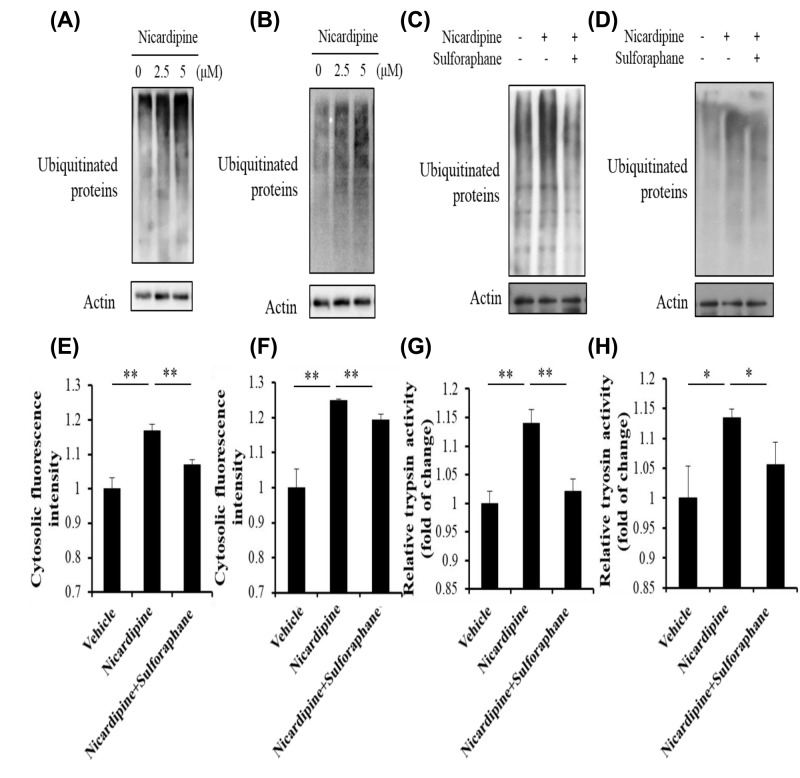
Nicardipine activated trypsinogen through proteasome inhibition AR42J cells (A,C,E and G) or primary pancreatic cells (B,D,F and H) were treated with nicardipine at indicated concentration with or without sulforaphane 10 μM for 6 h (primary cells for 1 h). Cells lysate were subjected to Western blotting using indicated antibodies (**A**–**D**) or trypsin activity assay (**G** and**H**). Cells were stained with fluo-4AM (1 μM), and then fluorescence intensities were assayed by FACS (**E** and **F**).

It has been reported that L-type calcium channel blocker verapamil could induce proteasome inhibition [30]. Moreover, proteasome inhibitors are reported to cause acute pancreatitis in clinic [28,29]. So, we detected the effect of nicardipine on proteasome. As we expected, nicardipine caused proteasome inhibition as the level of ubiquitinated protein increased under this compound treatment in AR42J cells and primary pancreatic acinar cells (Figure 7A,B). Since proteasome activity is very important for cell survival, we then investigated the relationship between proteasome inhibition and acinar cells injury induced by nicardipine. Proteasome activator sulforaphane was employed. As shown here, sulforaphane reversed the effect of nicardipine on proteasome activity in AR42J cells and primary pancreatic acinar cells (Figure 7C,D). After the recovery on the proteasome activity, the elevation in cytosolic calcium as well as trypsin activity was diminished by sulforaphane (Figure 7E–H). Meanwhile, sulforaphane alone didn’t affect cytosolic calcium and trypsin activity (Supplementary Figure S4A–D).

### Nicardipine induced proteasome inhibition depended on CaMKII

**Figure 8:**
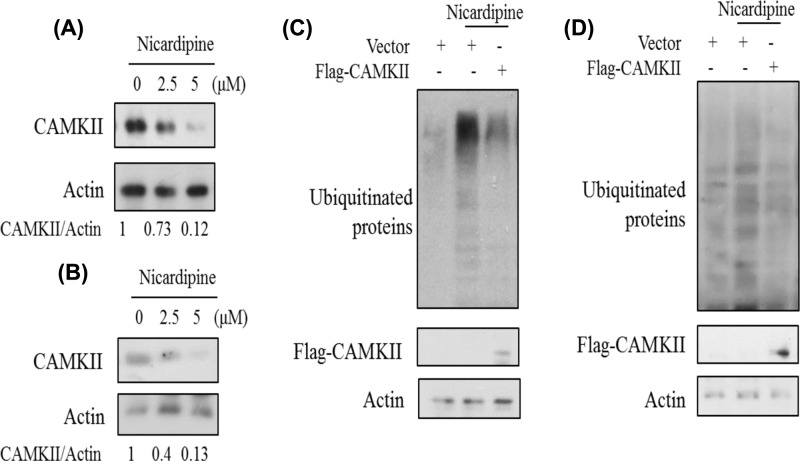
Nicardipine induced proteasome inhibition depended on CaMKII AR42J cells (**A** and **C**) or primary pancreatic acinar cells (**B** and **D**) were treated with nicardipine at indicated concentration (A and B), transfected with or without the CaMKII expression vector (C and D). Cell lysates were subjected to Western blotting using indicated antibodies. The bands (A and B) were quantified by ImageJ software as mean from two independent sets of experiments.

Previous report suggested that protein degradation was regulated by CaMKII during the memory reconsolidation process [33]. We questioned whether nicardipine-induced proteasome inhibition was mediated by CaMKII. First, the fluctuation in the protein level of CaMKII under nicardipine was measured. It was found that nicardipine indeed lead to the down-regulation in CaMKII expression (Figure 8A,B). Consistently, ectopic expression of CaMKII ameliorated the proteasome inhibition by nicardipine (Figure 8C,D). Meanwhile, CaMKII alone didn’t influence the level of ubiquitinated protein (Supplementary Figure S4E,F). The phosphorylation level of CaMKII is an indication of its activity. Nicardipine increased cytosolic calcium that should activate CaMKII. Therefore, the level of phosphorylated CaMKII was also detected. However, phosphorylated CaMKII decreased (Supplementary Figure S5).

## Discussion

The results showed that nicardipine could increase cathepsin B activity that caused trypsinogen activation, but higher concentration of nicardipine or prolonged treatment had an opposite effect. Besides, cytosolic calcium up-regulation induced by low dose nicardipine depended on proteasome dysfunction. Increased cytosolic calcium from ER induced by nicardipine resulted in the release and activation of cathepsin B. Meanwhile, calcium chelator inhibited cathepsin B as well as trypsinogen activation. Consistently, proteasome activator protected acinar cells from injury induced by nicardipine. Moreover, proteasome inhibition caused by nicardipine depended on CaMKII. Therefore, CaMKII down-regulation/proteasome inhibition/cytosolic calcium up-regulation/cathepsin B activation/trypsinogen activation axis was present in pancreatic acinar cells under nicardipine treatment.

In our study, another L-type calcium channel blocker, verapamil, was also detected. The results showed that verapamil at a relative low concentration or with short time treatment also induced cytosolic calcium, trypsin and cathepsin B (Supplementary Figure S6). Therefore, it might be common for L-type calcium channel blockers to induce acinar cells injury.

CaMKII activated proteasome through phosphorylation of proteasome regulatory subunit Rpt6 [34]. However, CaMKII under nicardipine treatment in the present study was found to inhibit proteasome that needs to be investigated further. Phosphorylated CaMKII that is the indicator of cytosolic calcium decreased under nicardipine. The phenomenon is uncommon and also needed to be illustrated later.

Our research illustrated that increased calcium/cathepsin B activation/trypsinogen activation might be one of the mechanisms underlying acute pancreatitis induced by the proteasome inhibitor Bortezomib or Ixazomib in patients with multiple myeloma. Thus, cytosolic calcium or cathepsin B could be considered to be the important point to ameliorate acute pancreatitis in patients treated with proteasome inhibitor. Of note, Bortezomib- or Ixazomib-induced acute pancreatitis was observed in elderly patients that suggested that proteasome inhibitors promote trypsinogen activation related to aging [27–29].

Proteasome is well known responsible for various misfolded proteins including Mfn1 and Mfn2 mitochondrial fusion proteins. In the endoplasmic reticulum (ER), many secretory and transmembrane proteins are folded during synthesis and checked for the correct folding [35]. Misfolded proteins are eventually retro-translocated into the cytosol for degradation by the proteasome [36]. So, the proteasome inhibition would lead to ER stress that might occur under nicardipine.

The L-type calcium channel blocker, nicardipine, which was initially thought to prevent extracellular calcium from embedding in cells, increased the total level of cytosolic calcium in AR42J cells. It was known that the resource of cytosolic calcium consisted of two major parts: exogenous and endogenous from mitochondrial, ER or lysosome calcium storage [37,38]. The up-regulation of cytosolic calcium was probably the outcome of changes in extracellular and intracellular calcium storage triggered by nicardipine. Since exogenous calcium through the L-type channel was blocked, together with the report that another calcium channel blocker verapamil could induce proteasome inhibition that might cause ER stress [30], nicardipine probably promoted calcium release from the ER in AR42J cells. Of note, extracellular calcium flux into cells could also be mediated by other types of calcium channel.

Recently, it was found that calcium leaked from intracellular storage, especially the ER, and then induced extracellular calcium flux into cells, leading to trypsinogen activation and secretion [39,40]. Therefore, the concept that store-operated calcium channels (CRAC channels) was established, and the relative blocker was considered to be a promising therapeutic approach [39,40]. Meanwhile, our study found that CRAC channel blocker inhibited cytosolic calcium induced by nicardipine. Therefore CRAC channels might be the targets of nicardipine.

Different from AR42J cells, primary pancreatic acinar cells do not possess voltage-gated calcium channels [39]. However, nicardipine had similar effects in both types of cells, indicating that L-type calcium channels might not be so important for intracellular trypsinogen activation induced by nicardipine. These results were consistent with the phenomenon found in a previous report [39].

Some voltage-dependent channel blockers have demonstrated protective effects in experimental acute pancreatitis [17,18]. However, in the present study, we found that nicardipine caused transient increment in cellular trypsin and amylase activity. Thus, the dose effect should not be ignored in calcium channel blocker-based drug design for acute pancreatitis treatment. Some voltage-gated channel blockers were found to ameliorate acute pancreatitis, while the opposite effect could also be observed under these blockers’ treatment [19]. These seemingly contradictory results might be due to the dual effect of channel blockers on trypsinogen activation. It should be noted that voltage-dependent calcium channel blockers might not be the best choice for hypertensive patients with a history of pancreatitis.

Among the various voltage-gated calcium channels, the L-type calcium channel was predominantly involved in depolarization to induce amylase secretion in AR42J cells [41]. Intracellular trypsin activity up-regulation might be partly due to blockage in secretion. Taken together, like amylase the secretion of trypsin under nicardipine treatment might also be inhibited.

In the present study, nicardipine-induced trypsinogen activation was mediated by cathepsin B. Meanwhile, cathepsin B was released into the cytosol, which was related to its increased activity. Here, we showed the direct visualized evidence for the effect on cathepsin B exerted by cytosolic calcium, which was rarely observed in previous studies. Furthermore, we show that in the development of acute pancreatitis, trypsin and cathepsin B induced the release of contents in the granule co-localized lysosome into the cytosol. The different mechanism for this leakage was revealed by a previous study that suggested that cathepsin B activated trypsinogen and was then released into the cytosol, in a manner mediated by trypsinogen [32]. Nevertheless, this phenomenon was not addressed in the present study.

## Conclusion

In conclusion, the present study suggested that the L-type calcium channel blocker nicardipine had dual effects on trypsin. Low dose or short time treatment with nicardipine blocked the proteasome via CaMKII and increased the level of cytosolic calcium from ER, leading to the activation of cathepsin B and subsequently, trypsinogen activation. However, the role of L-type calcium channels in acute pancreatitis might vary with the activity of calcium channels.

## Supporting information

**Supplementary Figure S1:** 

**Supplementary Figure S2:** 

**Supplementary Figure S3:** 

**Supplementary Figure S4:** 

**Supplementary Figure S5:** 

**Supplementary Figure S6:** 

## Abbreviations

DMEM: Dulbecco’s modified Eagle’s medium
ER: endoplasmic reticulum
FBS: fetal bovine serum
