# Supplementary material for: CaMKII/proteasome/cytosolic calcium/cathepsin B axis was present in tryspin activation induced by nicardipine
Source: Biosci Rep. 2019 Jul 2;39(7):BSR20190516. doi: 10.1042/BSR20190516 (PMC6603279; doi:10.1042/BSR20190516)
Supplement: Supplementary file 1 [file BSR20190516_Supp1.pdf]

Figure S1

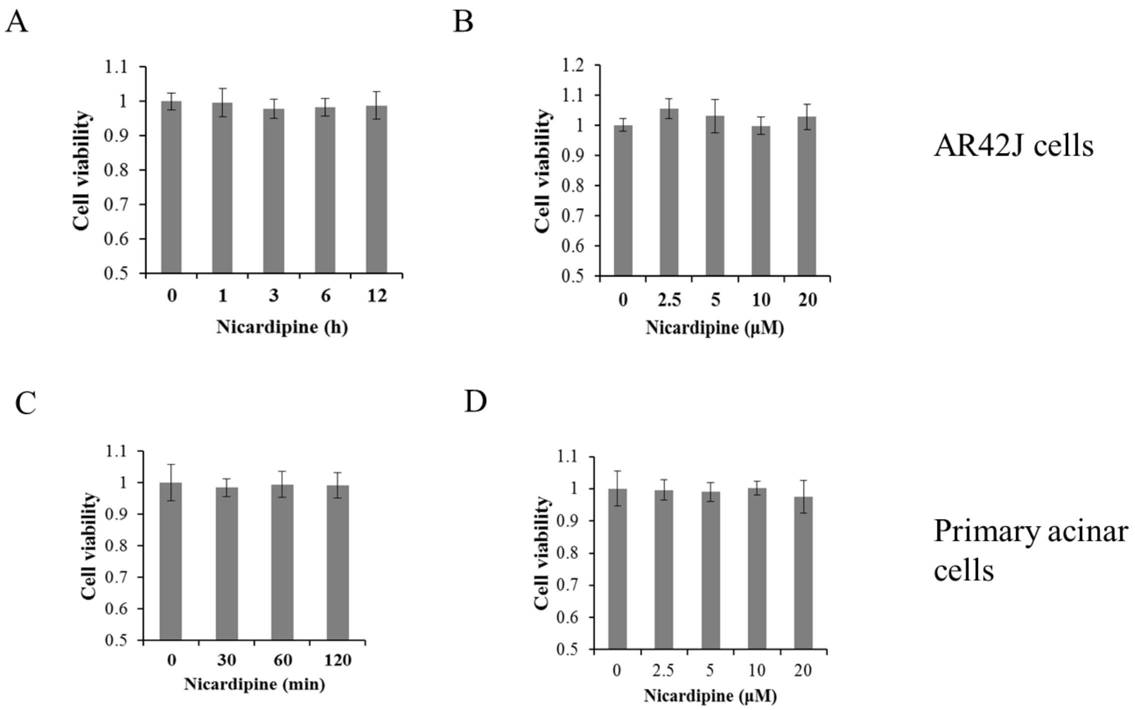
A
B
C
D
Cell viability
Nicardipine (h)
0
1
3
6
12
Nicardipine (μM)
0
2.5
5
10
20
Nicardipine (min)
0
30
60
120
AR42J cells
Primary acinar cells

Figure S2

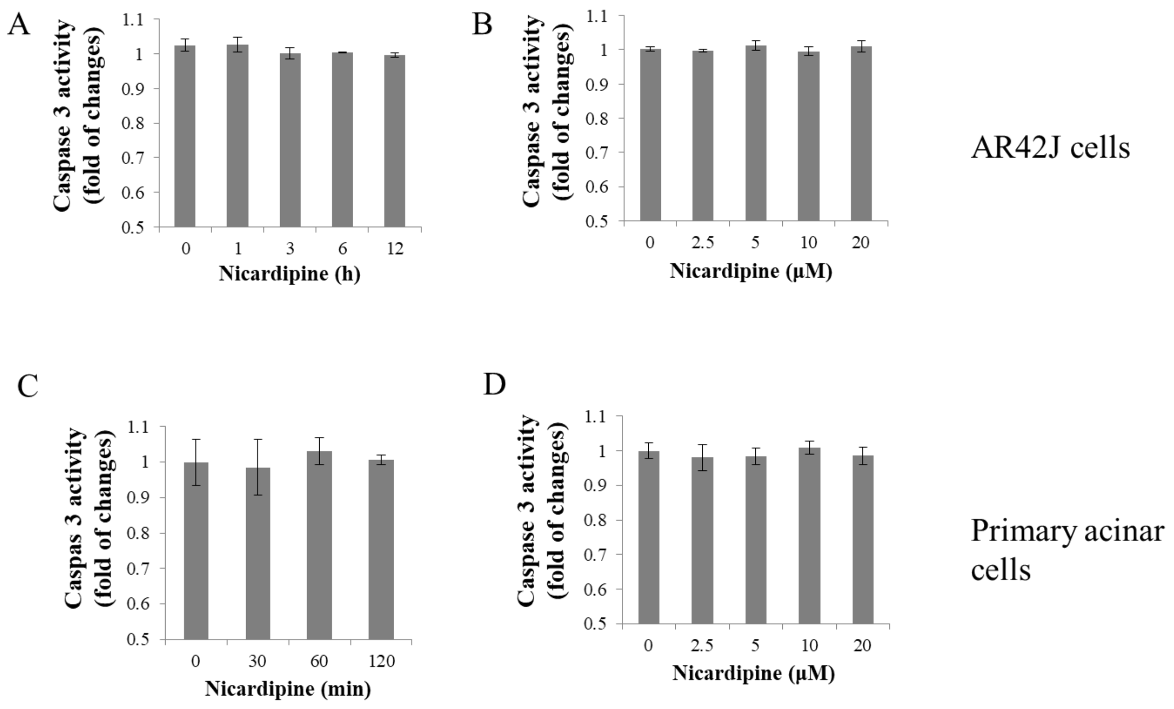
A
Caspase 3 activity (fold of changes)
Nicardipine (h)
B
Caspase 3 activity (fold of changes)
Nicardipine (μM)
AR42J cells
C
Caspas 3 activity (fold of changes)
Nicardipine (min)
D
Caspase 3 activity (fold of changes)
Nicardipine (μM)
Primary acinar cells

Figure S3

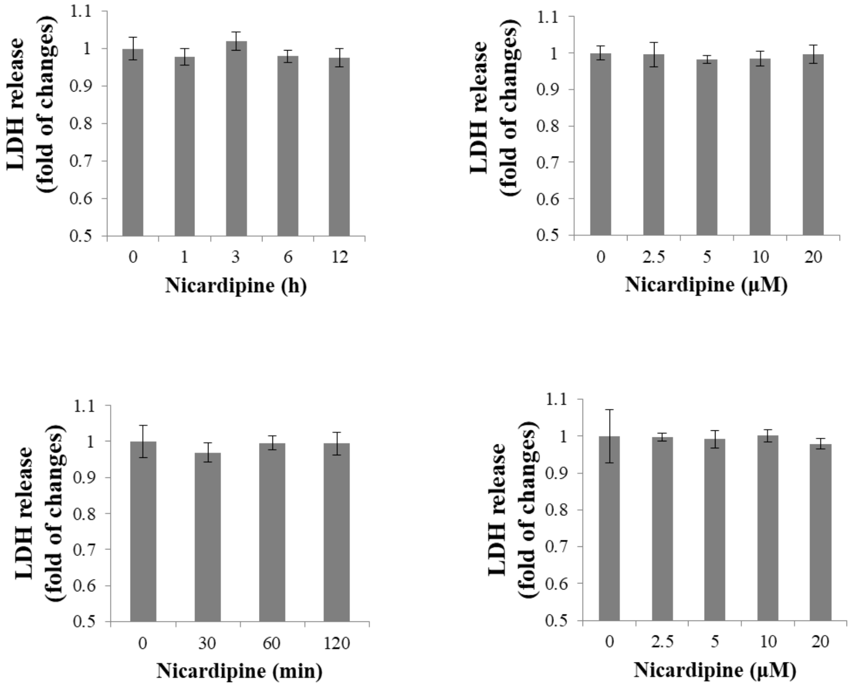

AR42J cells

Primary acinar cells

Figure S4

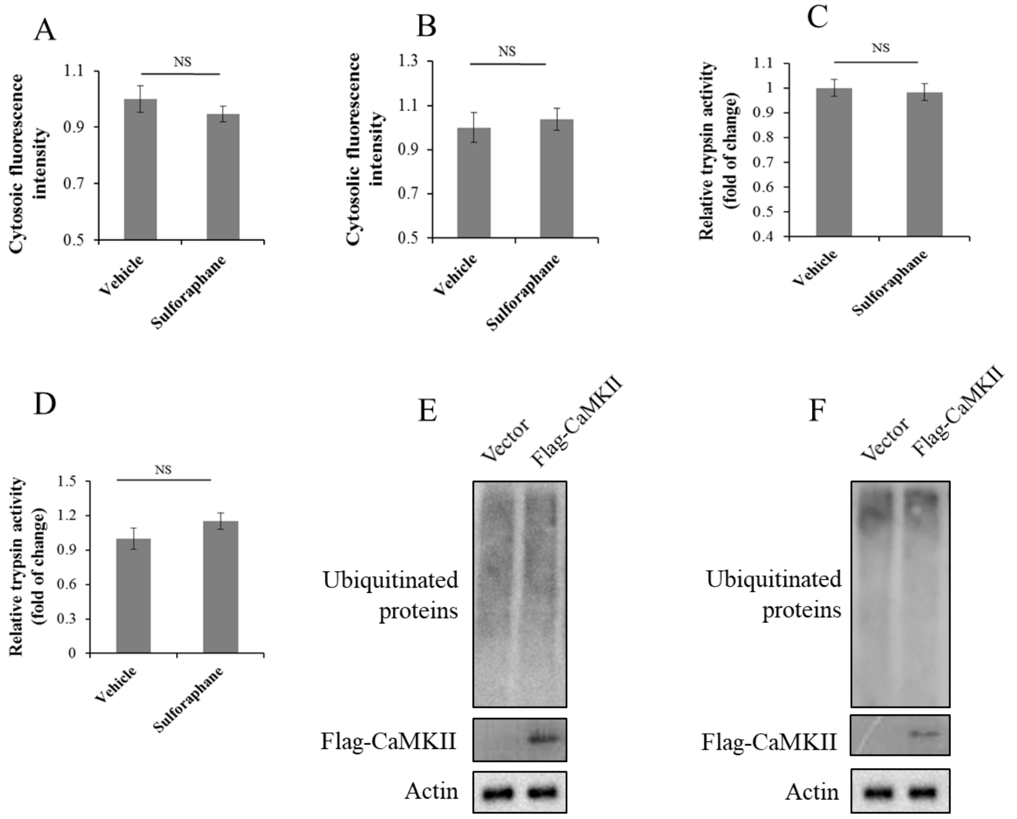

A
NS
Cytosoic fluorescence intensity
1.1
0.9
0.7
0.5
Vehicle
Sulforaphane
B
NS
Cytosolic fluorescence intensity
1.3
1.1
0.9
0.7
0.5
Vehicle
Sulforaphane
C
NS
Relative trypsin activity (fold of change)
1.1
1
0.9
0.8
0.7
0.6
0.5
0.4
Vehicle
Sulforaphane
D
NS
Relative trypsin activity (fold of change)
1.5
1.2
0.9
0.6
0.3
0
Vehicle
Sulforaphane
E
Vector
Flag-CaMKII
Ubiquitinated proteins
Flag-CaMKII
Actin
F
Vector
Flag-CaMKII
Ubiquitinated proteins
Flag-CaMKII
Actin

Figure S5

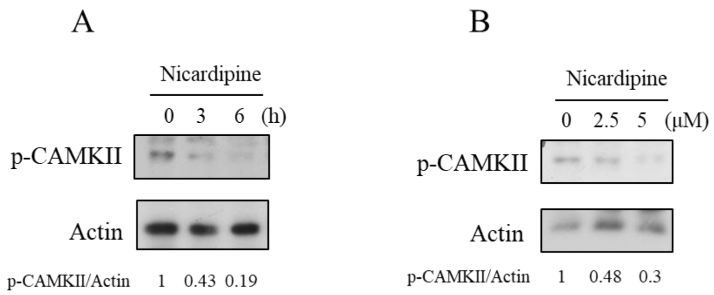
A
Nicardipine
0 3 6 (h)
p-CAMKII
Actin
p-CAMKII/Actin 1 0.43 0.19
B
Nicardipine
0 2.5 5 (μM)
p-CAMKII
Actin
p-CAMKII/Actin 1 0.48 0.3

Figure S6

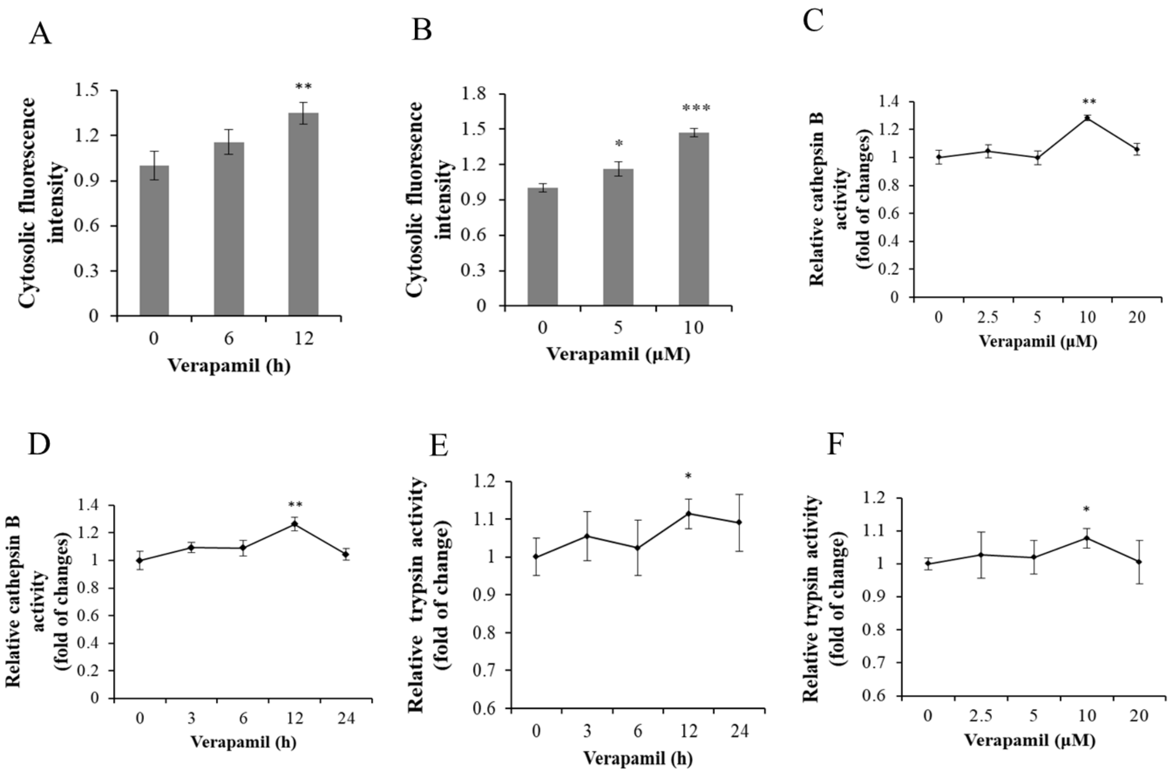
A
Cytosolic fluorescence intensity
Verapamil (h)
B
Cytosolic fluoresence intensity
Verapamil (μM)
C
Relative cathepsin B activity (fold of changes)
Verapamil (μM)
D
Relative cathepsin B activity (fold of changes)
Verapamil (h)
E
Relative trypsin activity (fold of change)
Verapamil (h)
F
Relative trypsin activity (fold of change)
Verapamil (μM)
